# Predictors of antiproliferative effect of lanreotide autogel in advanced gastroenteropancreatic neuroendocrine neoplasms

**DOI:** 10.1007/s12020-019-02086-6

**Published:** 2019-09-25

**Authors:** Faidon-Marios Laskaratos, Eleni Armeni, Heer Shah, Maria Megapanou, Dimitrios Papantoniou, Aimee R Hayes, Shaunak Navalkissoor, Gopinath Gnanasegaran, Conrad von Stempel, Edward Phillips, Myles Furnace, Lukasz Kamieniarz, Margarita Kousteni, Tu Vinh Luong, Jennifer Watkins, Dalvinder Mandair, Martyn Caplin, Christos Toumpanakis

**Affiliations:** 1 Centre for Gastroenterology, Neuroendocrine Tumour Unit, ENETS Centre of Excellence, Royal Free London NHS Foundation Trust, London, UK; 2 UCL Medical School, London, UK; 3 Department of Nuclear Medicine, Royal Free London NHS Foundation Trust, London, UK; 4 Department of Radiology, Royal Free London NHS Foundation Trust, London, UK; 5 Academic Centre for Cellular Pathology, Royal Free London NHS Foundation Trust, London, UK

**Keywords:** Lanreotide autogel, Neuroendocrine tumour, Somatostatin analogue, Neuroendocrine neoplasm

## Abstract

**Purpose:**

The antiproliferative properties of lanreotide autogel (LAN) in gastroenteropancreatic neuroendocrine neoplasms (GEP NENs) were demonstrated in the CLARINET study. However, there is limited literature regarding factors that affect progression-free survival (PFS) in patients with GEP NENs treated with LAN.

**Methods:**

We identified a total of 191 treatment-naive patients with advanced GEP NENs and positive SSTR uptake on imaging (Octreoscan or ^68^Gallium DOTATATE Positron Emission Tomography [^68^GaPET]) who received first-line LAN monotherapy, albeit at various starting doses (60, 90 or 120 mg/month). A group of 102 patients who initiated treatment at the standard dose of 120 mg/month were included in the study and further evaluated by univariate and multivariate analyses to identify predictors of PFS.

**Results:**

The location of tumour primary was in the small bowel in 63 (62%), pancreas in 31 (30%) and colon/rectum in 8 patients (8%). The tumours were well-differentiated, and the majority were grade 1 (52%), or 2 (38%). About 60% of cases had progressive disease at the time of treatment initiation. Most patients with available pretreatment nuclear medicine imaging (Octreoscan or ^68^Ga PET) had a Krenning score of 3 (44%) or 4 (50%). The median PFS for the entire cohort was 19 months (95% CI 12, 26 months). The univariate analysis demonstrated that grade 2 tumours, progressive disease at baseline and metastatic liver disease were associated with a significantly shorter PFS, while other evaluated variables did not affect PFS at a statistically significant level. However, at multivariate analysis only the tumour grade remained statistically significant.

**Conclusions:**

The current study showed that, of many evaluated variables, only the tumour grade was predictive of PFS duration and this should be considered during patient selection for treatment.

## Introduction

Neuroendocrine neoplasms (NENs) (term encompassing both well-differentiated neuroendocrine tumours [NETs] and poorly differentiated neuroendocrine carcinomas) are rare neoplasms arising from cells of the diffuse endocrine system. Although they share some common features, there is significant heterogeneity in their clinical presentation, behavioural characteristics and natural history [1, 2].

Somatostatin analogues (SSAs) are synthetic derivatives of human somatostatin and are considered the mainstay of therapy for both symptomatic management and tumour growth. The antiproliferative role of long-acting SSAs was confirmed in the PROMID [3] and CLARINET [4] studies. The CLARINET trial demonstrated the antiproliferative effects of lanreotide autogel (LAN) in patients with metastatic, well- or moderately differentiated, non-functional enteropancreatic NETs [4]. An open label extension of the CLARINET study provided additional information regarding the antiproliferative effects of LAN, which was associated with a median progression-free survival (PFS) of 30.8 months. In addition, patients in the placebo group with progressive disease who then switched to open label LAN had a median PFS of 14 months (95% CI 10.1, not reached) [5].

Currently there is limited literature on factors that predict PFS in patients with NENs receiving SSAs. A recent study from our centre focused on predictive factors affecting PFS in patients with advanced NENs treated with octreotide LAR and identified several predictors of response [6]. The present study aimed to determine the median PFS and evaluate factors affecting PFS in a cohort of patients with gastroenteropancreatic (GEP) NENs treated with LAN 120 mg/month as first-line monotherapy in a tertiary centre.

## Materials and methods

### Patients

A total of 191 patients were initially identified in our database who had well-differentiated GEP NENs and received first-line LAN monotherapy, albeit at different starting dosing regimens. All cases had a confirmed histopathological diagnosis and were treated in our centre between 2000 and 2016. These patients were treatment-naive and had nuclear medicine imaging studies confirming positive SSTR uptake (Octreoscan or ^68^Ga-PET) prior to commencing therapy. LAN injections were administered at the standard dose of 120 mg/ 28 days in 102 patients (53% of cases), who were included in the study and evaluated further by univariate and multivariate analyses to identify predictors of response to therapy.

Some of the LAN-treated GEP NET patients had been started on LAN before publication of the CLARINET study data and therefore lower starting doses were used (either 60 mg/28 days [18%] or 90 mg/28 days [29%]). These patients were excluded from further analyses to allow the study of a more homogeneous patient cohort that received the same starting dose of LAN.

### Study design

A retrospective review of electronic patient records was performed. Data collected comprised patient demographics (age, gender) and other baseline information (medical comorbidities, treatment indication for SSA), tumour characteristics (location of primary, primary resection, functional status, grade, presence of carcinoid heart disease [CHD], presence of liver or extrahepatic metastases, SSTR uptake on Octreoscan or ^68^Ga PET imaging and PFS), as well as biochemical data at baseline (serum chromogranin A [CgA] and 5-hydroxyindoleacetic acid [5-HIAA] levels prior to SSA therapy). Patients with active secondary malignancy were excluded from the study.

NETs were classified and graded according to the WHO 2019 classification [7]. The extent of liver involvement was assessed using the following scoring system: (0) absence of liver metastases, (1) volume of liver metastases affecting <25% of hepatic parenchyma, (2) volume of liver metastases affecting 25–50% of hepatic parenchyma and (3) volume of liver disease >50%.

SSTR uptake on nuclear medicine imaging studies (Octreoscan or ^68^Ga PET imaging) was classified according to the Krenning scale (1) uptake less than background liver, (2) uptake equal to background liver, (3) uptake greater than background liver, (4) uptake greater than spleen. SSTR uptake was evaluated using Octreoscan or ^68^Ga PET and pretreatment nuclear medicine studies were available for review in 76% (78/102) of patients. Baseline (pretreatment) Octreoscan was not available for evaluation in 24% of cases, usually because it was performed at another institution. However, this had been reviewed prior to LAN commencement in our multidisciplinary tumour board and had demonstrated positive SSTR uptake. Thus, all the included patients had positive SSTR-uptake status before treatment, but the Krenning score could only be precisely evaluated in 76% of cases. Although the Krenning score was originally used and validated for Octreoscan, a common approach for characterising uptake on ^68^Ga-PET is to use a ‘modified’ Krenning score, where the same qualitative approach (comparison with the liver uptake) is applied to SSTR-PET imaging [8, 9].

Radiological assessment was based on cross-sectional imaging (CT or MRI) in 4–6 month intervals, using the revised RECIST (version 1.1) criteria to determine disease progression [10]. A number of variables were assessed as potential predictive factors of response to treatment with LAN. These included:

1. Demographic data: age (<65 or ≥65), sex (male or female) and comorbidity (<2 or ≥2 medical comorbidities).
2. Tumour-related characteristics: primary site (small intestinal, pancreatic or colorectal), tumour grade (1 or 2 using the WHO 2019 classification; G3 and poorly differentiated neoplasms were excluded from the study), functionality (presence or not of functional symptoms) and extent of metastatic spread, including both liver disease burden (none, <25%, 25–50% or >50%) and extrahepatic sites (<2 or ≥2), which were also evaluated separately as predictors of response (i.e., presence of skeletal, peritoneal, lung, breast and lymph node metastases above and below the diaphragm). In addition, we evaluated SSTR uptake (using the Krenning scale), disease status (stable or progressive disease; progressive status at baseline was defined as disease progression during the previous 12 months before LAN initiation) and association with CHD (for small intestinal NENs).
3. Biomarker levels: CgA and urinary 5-HIAA (for small intestinal NENs) (using the following categories: normal levels, <5 times, 5–10 times or >10 times the upper limit of the normal range).

Their relevance as predictors of response to LAN therapy was assessed by univariate and multivariate analyses.

Tumour biomarker levels (CgA and urinary 5-HIAA for small intestinal NETs) before (within 3 months of) treatment initiation and 12 months after starting LAN were also recorded to monitor biochemical response to therapy.

### Statistical analysis

Non-parametric Kaplan–Meier techniques were used to evaluate PFS, including the median and associated 95% confidence intervals, in strata defined by various explanatory variables. PFS was defined as the period from the initiation of treatment (LAN 120 mg/monthly) until disease progression or death. A semi-parametric Cox regression model was used to perform multivariate analysis, using variables identified as statistically significant by univariate analysis. A *p*-value < 0.05 was considered statistically significant. Statistical analysis was performed using SPSS version 25.

## Results

### Patient characteristics

A total of 102 patients were included in this analysis. The mean age (±SD) of the patient population was 62 ± 12 years. The male to female ratio in the study was 1.2:1. The location of tumour primary was in the small bowel in 63 (62%), pancreas in 31 (30%) and colon/rectum in 8 patients (8%). The histological grade was G1 in 53 (52%), G2 in 39 (38%) and not available in 10 patients (10%). The study population included 17 patients (17%) with no liver disease, 66 patients (65%) with liver disease volume <25%, 12 patients (12%) with 25–50% liver tumour burden and 7 (7%) with a liver disease volume >50%. The primary tumour was resected before commencement of LAN in 53 patients (52%), all of whom had residual disease and were commenced on SSA therapy after surgery. The commonest sites of extrahepatic disease were the bones (present in 18% of cases), lymph nodes above (13%) and below the diaphragm (56%), peritoneum (28%), followed by the lung (5%) and breast (1%). Indications for commencement of LAN included functional symptoms in 38 (37%) and radiological progression in 61 (60%). Of these patients, 20 had a combination of functional symptoms and radiological progression. There were also 17 patients (17%) who were asymptomatic with stable disease and initiated on treatment based on the antiproliferative effects of SSAs. The patients in the latter group were diagnosed after 2014, when data from the CLARINET study emerged and supported the antiproliferative effects of LAN in non-functioning GEP NETs with stable disease status. The indication for treatment initiation was not clearly documented in six cases. Nuclear medicine studies prior to initiation of SSA treatment were available for review in 76% of cases (78/102). SSTR uptake was evaluated using Octreoscan in 45% (35/78) and 68Ga PET in 55% (43/78) of those cases. Most evaluated patients had a Krenning score of 3 (44%) or 4 (50%).

A summary of patient baseline characteristics is provided in Table 1.

**Table 1:** Summary of baseline characteristics in a cohort of 102 patients with advanced low and intermediate grade neuroendocrine tumours treated with standard doses of lanreotide autogel as first-line monotherapy

	*N* = 102 (%)
Sex	
Male	55 (54)
Female	47 (46)
Indication for treatment	
Functional symptoms	38 (37)
Radiological progression	61 (60)
Asymptomatic and stable disease	17 (17)
Not clearly documented	6 (6)
Location of primary	
Small bowel	63 (62)
Pancreas	31 (30)
Colon & Rectum	8 (8)
Grade	
G1	53 (52)
G2	39 (38)
Unknown	10 (10)
Functional Status	
Functional	38 (37)
Non-functional	64 (63)
Carcinoid heart disease	
Yes	7 (7)
No	95 (93)
Chromogranin A	
Normal	17 (17)
Up to 5 times higher than upper limit of normal	13 (13)
Between 5–10 times higher than upper limit of normal	13 (13)
>10 times upper limit of normal	16 (16)
Unknown	43 (42)
Urinary 5-HIAA	
Normal	19 (19)
Up to 5 times higher than upper limit of normal	11 (11)
Between 5–10 times higher than upper limit of normal	4 (4)
>10 times upper limit of normal	11 (11)
Unknown	57 (56)
Primary tumour resection	
Yes	53 (52)
No	49 (48)
Krenning Score	
Not available	24 (24)
1	0 (0)
2	5 (5)
3	34 (33)
4	39 (38)

### Progression-free survival

**Fig. 1:**
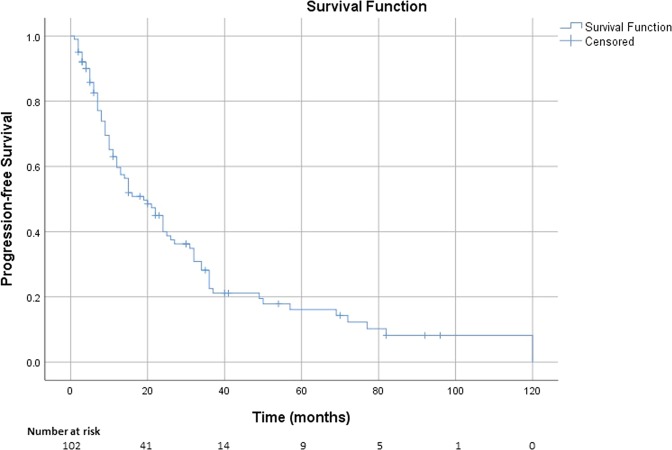
Kaplan–Meier estimate of the survival function for the time to tumour progression for the entire cohort of 102 patients. The median PFS was 19 months (95% CI 12, 26 months)

The median PFS for the entire cohort was 19 months (95% CI 12, 26 months) (Fig. 1).

### Univariate analysis of factors influencing progression-free survival

The univariate analysis demonstrated that grade 2 tumours, progressive disease at initiation of treatment and metastatic liver disease were predictive factors of a significantly shorter PFS.

#### Tumour grade

**Fig. 2:**
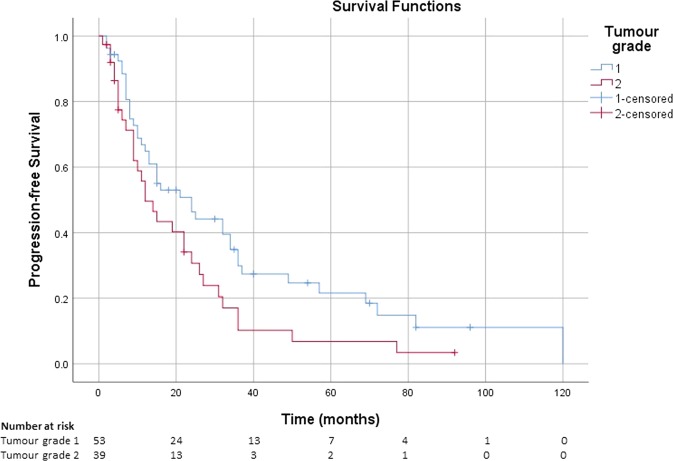
Kaplan–Meier estimate of the survival function for the PFS in patients with advanced NETs receiving lanreotide autogel, stratified by tumour grade at diagnosis

The median PFS was 24 months (95% CI: 11, 37 months) for patients with G1 tumours, while patients with G2 tumours had a median PFS of 12 months (95% CI: 6, 18 months) (Fig. 2).

#### Disease status at initiation of treatment

**Fig. 3:**
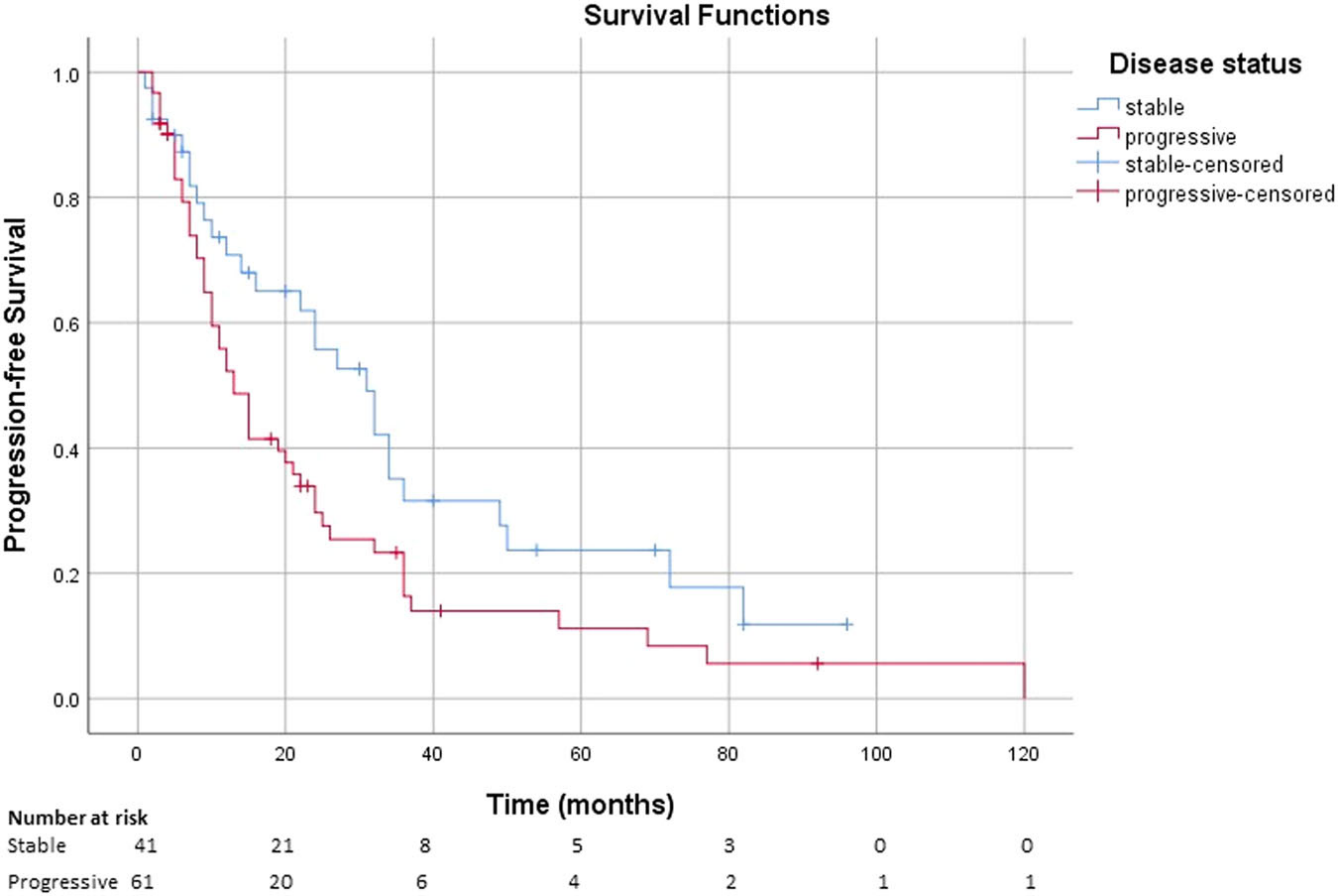
Kaplan–Meier estimate of the survival function for the PFS in patients with advanced NETs receiving lanreotide autogel, stratified by disease status (progressive vs. stable) at initiation of treatment

The median PFS was 31 months (95% CI: 21, 41) in patients with stable disease, while patients with progressive disease had a median PFS of 13 months (95% CI: 9, 17) (Fig. 3).

#### Liver involvement

**Fig. 4:**
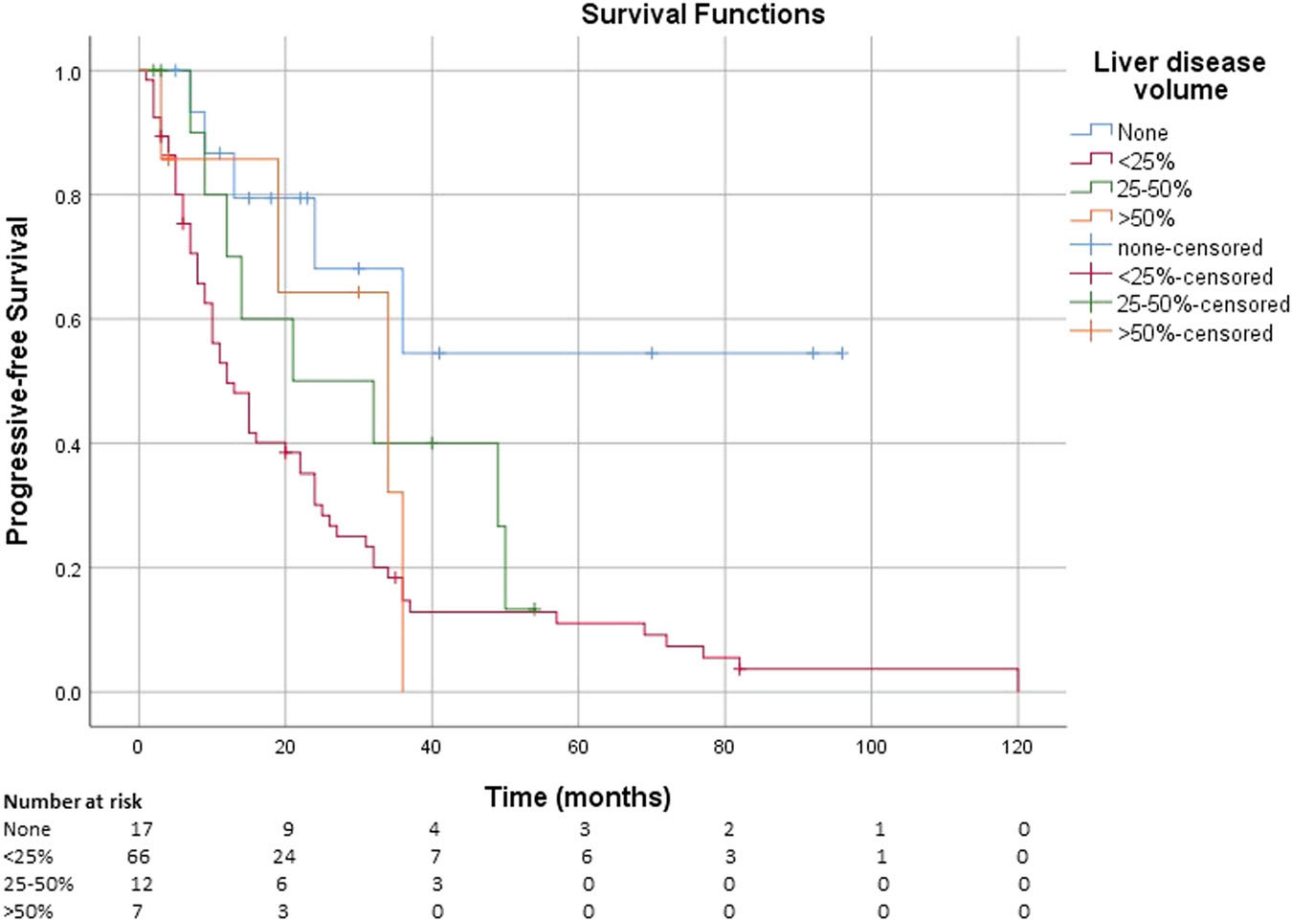
Kaplan–Meier estimate of the survival function for the PFS in patients with advanced NETs receiving lanreotide autogel, stratified by % liver disease volume at diagnosis

The median PFS was not reached for patients without liver involvement, while it was 12 months (95% CI: 8, 16 months) for those with liver disease <25%, 21 months (95% CI: 0, 49 months) for those with liver tumour burden 25–50%, and 34 months (95% CI: 11, 60 months) for patients with liver disease >50% (Fig. 4).

In contrast, age, gender, primary tumour site, presence of CHD, other metastases, resection of the tumour primary, SSTR uptake, baseline CgA and urinary 5-HIAA levels were not found to be predictive factors affecting response to treatment.

The results of the univariate analysis are shown in Table 2.

**Table 2:** Univariate analysis of predictive factors of response to standard doses of first-line lanreotide autogel in a cohort of 102 advanced low and intermediate grade neuroendocrine tumours

Variable	Category	HR	95% CI (lower)	95% CI (upper)	*p*-value
Age	<65	(ref)			
	≥65	1.28	0.81	2.02	0.29
Sex	Male	(ref)			
	Female	0.87	0.57	1.41	0.64
Medical comorbidities	<2	(ref)			
	≥2	1.31	0.72	2.40	0.38
Tumour primary	Small bowel	(ref)			
	Pancreas	0.91	0.54	1.53	0.72
	Colon and rectum	1.53	0.72	3.25	0.27
Tumour grade	G1	(ref)			
	G2	1.60	0.99	2.57	0.049^a^
Krenning score	2	(ref)			
	3	0.39	0.09	1.74	0.22
	4	0.39	0.09	1.72	0.21
Primary tumour resection	No	(ref)			
	Yes	1.20	0.75	1.91	0.44
Liver disease volume	None	(ref)			
	<25%	4.19	1.68	10.46	0.002^a^
	25–50%	2.56	0.83	7.85	0.10
	>50%	2.79	0.75	10.48	0.13
Tumour functionality	No	(ref)			
	Yes	1.49	0.94	2.35	0.09
Disease status at initiation of treatment	Stable	(ref)			
	Progressive	1.64	1.02	2.64	0.04^a^
Extrahepatic metastases	<2 sites	(ref)			
	≥2 sites	1.07	0.67	1.70	0.78
Lymphadenopathy above the diaphragm	No	(ref)			
	Yes	0.69	0.33	1.44	0.32
Lymphadenopathy below the diaphragm	No	(ref)			
	Yes	0.83	0.52	1.31	0.42
Skeletal metastases	No	(ref)			
	Yes	1.48	0.81	2.70	0.20
Lung metastases	No	(ref)			
	Yes	1.49	0.54	4.10	0.44
Peritoneal metastases	No	(ref)			
	Yes	0.74	0.45	1.22	0.24
Breast metastases	No	(ref)			
	Yes	0.70	0.10	5.03	0.72
Carcinoid heart disease^b^	No	(ref)			
	Yes	0.41	0.13	1.31	0.13
Chromogranin A levels	Normal	(ref)			
	<5× the upper limit of normal	0.97	0.43	2.17	0.93
	5–10× the upper limit of normal	0.76	0.33	1.74	0.52
	>10× the upper limit of normal	0.65	0.28	1.49	0.31
Urinary 5-HIAA levels^b^	Normal	(ref)			
	<5× the upper limit of normal	0.58	0.23	1.44	0.24
	5–10× the upper limit of normal	1.23	0.41	3.75	0.71
	>10× the upper limit of normal	0.36	0.12	1.14	0.08

^a^Denotes statistical significance (*p* < 0.05)

^b^Analysis restricted to small bowel NETs

### Multivariate analysis of factors influencing progression-free survival

The multivariate analysis demonstrated that grade 2 tumours (HR 1.64, 95% CI: 1.01, 2.67, *p* = 0.04) remained an independent predictor of shorter PFS, while the presence of progressive disease and liver disease volume did not retain statistical significance. The results of the multivariate analysis are shown in Table 3.

**Table 3:** Multivariate analysis of predictors of response to first-line monotherapy with standard doses of lanreotide autogel in a cohort of 102 patients with advanced low/intermediate grade neuroendocrine tumours

Variable	Category	HR	95% CI (lower)	95% CI (upper)	*p*-value
Liver disease volume	None	(ref)			
	<25%	0.39	0.10	1.48	0.17
	25–50%	1.30	0.47	3.60	0.62
	>50%	1.00	0.29	3.45	0.99
Disease status at initiation of treatment	Stable	(ref)			
	Progressive	1.49	0.87	2.52	0.14
Tumour grade	G1	(ref)			
	G2	1.64	1.01	2.67	0.04^*^

^*^indicates statistical significance

### Tumour biomarker responses

CgA levels were available for evaluation at baseline and 12 months after LAN initiation in 49 cases (48% of study population). CgA levels decreased by 54% on average and 26 of these patients (53%) had a significant (>50%) drop in their CgA levels.

In addition, urinary 5-HIAA levels were available for assessment at baseline and 12 months after LAN initiation in 12 patients (19%) with SINENs. Urinary 5-HIAA levels decreased by an average of 36% and six of these patients (50%) had a significant (>50%) drop in their urinary 5-HIAA levels.

## Discussion

The present study showed that the median PFS in a large institutional cohort of 102 treatment-naive patients with low/intermediate grade GEP NETs treated with first-line standard dose LAN monotherapy was 19 months. In the univariate analysis the response to treatment was adversely affected by hepatic tumour load, progressive disease status and tumour grade (grade 2) at initiation of treatment. However, at multivariate analysis only the tumour grade was independently associated with PFS duration. Other factors (age and gender, location of primary tumour, surgical resection of primary tumour, presence of extrahepatic metastases, SSTR uptake, baseline levels of CgA or urinary 5-HIAA) did not appear to be predictive of response to treatment. In a previous large retrospective study from our centre evaluating predictors of PFS in a cohort of 254 treatment naive patients with NET treated with octreotide LAR, the median PFS was 37 months and liver tumour burden, higher tumour grade (grade 2) and pancreatic primaries were predictive of a shorter PFS [6].

Other studies that evaluated the median PFS in patients on LAN treatment for the management of GEP or bronchial NETs are summarised in Supplementary File 1 (Table 1a, b) [4, 5, 11–21]. The PFS reported in our study is in agreement with other publications [4, 5, 13, 14, 16, 20, 21]. The CLARINET and CLARINET open label extension study demonstrated the efficacy of LAN monotherapy as a treatment modality of patients with GEP NETs of grade 1 or 2 (with Ki67 < 10%) [4, 5]. However, there is limited literature regarding predictors of LAN efficacy.

The present study demonstrated that tumour grade was the only predictive factor independently associated with PFS duration. G2 tumours were associated with a significantly shorter PFS and an HR of 1.64 (95% CI: 1.01, 2.67) (*p* = 0.04). In agreement with our findings, a Spanish multicentre study confirmed that lower Ki67 ranking was associated with a lower risk of disease progression (Ki67 ranking, HR: 1.17, 95% CI: 1.03, 1.33; *p*-value = 0.02) [11]. Similarly, in another study patients with Ki67 ≥5% demonstrated an up to 73.8% higher risk of disease progression on LAN treatment compared with patients diagnosed with Ki67 <5% at baseline [13].

In addition, our study showed an association between progressive disease status at initiation of LAN treatment and risk of further disease progression at univariate analysis, but this factor was no longer significant at the multivariate analysis. The median PFS of patients with progressive disease at baseline was 13 months (95% CI: 9, 17), while those patients with stable disease had a median PFS was 31 months (95% CI: 21, 41). These findings are in keeping with the CLARINET-OLE study, in which patients with progressive disease who received LAN had a median time to further progression of 14 months [5], and this was significantly shorter than the reported PFS for patients with stable disease, suggesting an inverse association between LAN efficacy and disease progression at initiation of treatment. However, our analysis did not identify this as an independent factor of LAN efficacy.

Furthermore, the presence of liver metastases was associated with a significantly shorter PFS at univariate analysis, since patients with <25% liver disease volume had a significantly shorter PFS compared with patients without liver metastases. However, there was absence of proportionality of the risk of progression in strata defined by the volume of liver disease and this factor was no longer statistically significant by multivariate analysis. Therefore, our study did not confirm the significance of liver tumour burden as an independent risk factor for progression on first-line LAN monotherapy. The predictive role of the hepatic tumour load in the response to SSA treatment has been assessed only in a few studies. In the PROMID trial [3], the only independent prognostic factor for prolonged time to progression or tumour-related death was a low hepatic tumour burden (≤10%). In the CLARINET trial [4], a shorter PFS was demonstrated in patients with hepatic tumour burden >25% although both groups received benefit with LAN. Further studies would be useful to define the impact of liver disease volume on LAN efficacy.

Limitations of this study include its retrospective design and the potential interpretation bias. Another limitation of our study is that the proportion of patients with high hepatic tumour burden at baseline was relatively small, and therefore this subgroup of patients is relatively under-represented. In addition, this study was conducted in a single tertiary referral centre and therefore our clinical practices and patient population may not be typical of those in smaller centres. Furthermore, most cases had small bowel primary tumours, which means that our findings might be less applicable to patients with pancreatic and colorectal NENs, that were smaller subgroups of our study. However, the primary tumour site did not appear to significantly affect PFS, although this may be subject to selection bias. Another limitation of this study is that the Krenning score was evaluated by Octreoscan in some patients and ^68^Ga-PET in others. A recent study analysing head-to-head comparisons of Krenning scores in Octreoscan and ^68^Ga-PET showed that the detection of SSTR-expressing disease (Krenning score ≥2) was significantly higher in ^68^Ga-PET compared with Octreoscan. In addition, the Krenning scores were higher in ^68^Ga-PET than in Octreoscan, but this was particularly an issue with small lesions (<2 cm), while Krenning scores were nearly equivalent with lesions greater than 5 cm [9]. Although this publication highlights differences in Krenning score assessments using these different imaging modalities, these were more prominent in small lesions. Our study included only patients with advanced (metastatic) disease and therefore most patients had larger lesions that would presumably eliminate many of these differences. However, this is certainly a limitation of this retrospective analysis.

In conclusion, our study population represents the largest cohort of patients in the literature with GEP NENs treated with LAN first-line monotherapy in a single institution and has provided additional information regarding the antiproliferative effects of LAN. Tumour grade appeared to be the only predictive factor independently associated with PFS duration in this analysis. Of course, the management of patients should be discussed within a multidisciplinary tumour board considering the specific characteristics of each individual case. Treatment naive patients with grade 2 GEP NETs selected for treatment with first-line long-acting SSAs should be monitored more closely to identify disease progression and initiate, if necessary, second-line therapeutic strategies at an early stage.

## Supplementary information

Supplementary File 1

## Data Availability

All data generated or analysed in this study are included in this article [and its supplementary information files].

## List of abbreviations

CgA: Chromogranin A
CHD: Carcinoid heart disease
CI: Confidence interval
GEP: Gastroenteropancreatic
5-HIAA: 5-hydroxyindoleacetic acid
HR: Hazard ratio
LAN: Lanreotide autogel
NEC: Neuroendocrine carcinoma
NEN: Neuroendocrine neoplasm
NET: Neuroendocrine tumour
PFS: Progression-free survival
SSA: Somatostatin analogue
