# Supplementary material for: Predictors of antiproliferative effect of lanreotide autogel in advanced gastroenteropancreatic neuroendocrine neoplasms
Source: Endocrine. 2019 Sep 25;67(1):233–42. doi: 10.1007/s12020-019-02086-6 (PMC6968981; doi:10.1007/s12020-019-02086-6)
Supplement: Supplementary file 1 [file 12020_2019_2086_MOESM1_ESM.pdf]

Supplementary File 1.

Title:

Predictors of Antiproliferative Effect of Lanreotide Autogel in Advanced Gastroenteropancreatic Neuroendocrine Neoplasms

Authors:

Faidon-Marios Laskaratos, Eleni Armeni, Heer Shah, Maria Megapanou, Dimitrios Papantoniou, Aimee R Hayes, Shaunak Navalkissoor, Gopinath Gnanasegaran, Conrad von Stempel, Edward Philips, Myles Furnace, Margarita Kousteni, Dalvinder Mandair, Martyn Caplin, Christos Toumpanakis

Corresponding author:

Dr Faidon-Marios Laskaratos MBBS, MSc, MRCP (UK), MRCP (London)

Centre for Gastroenterology, Neuroendocrine Tumour Unit, ENETS Centre of Excellence, Royal Free London NHS Foundation Trust, London, UK

e-mail: flaskaratos@gmail.com

Supplementary table 1a. Studies evaluating the PFS in patients treated with Lanreotide monotherapy								
**Author**	**Type of study**	**Sample size**	**Lanreotide regimen**	**Previous SSA treatment**	**Location of tumour**	**PFS**	**Liver disease**	**Predictors of PFS**
Caplin et al 2014 (4)	double blind randomized placebo controlled	101 cases LAN, 103 cases placebo<br>- 4% progressive disease at baseline	120mg LAN (4weekly) or placebo for a total of 96 weeks	84% no previous treatment	LAN subgroup: Pancreas 42%; Midgut 33%; Hindgut 11%; Unknown or other 15%	24 months: 65.1% (95% CI, 54.0 to 74.1)	HTL after treatment ≤25% 137 patients, >25% 67 patients	no data
Martin-Richard et al 2013 (11)	multicenter, open-label, single arm phase II study	30 patients with advanced, well-differentiated GEP, bronchopulmonary NET or neuroendocrine carcinoma	LAN 120mg (4weekly) for up to 92 weeks	surgery 23; SSA 6; RT 1; any 15	pancreas 26.7%; stomach 3.3%; small intestine 33.3%; large intestine 10%; bronchopulmonary 4%; unknown 4%	12.9 (95% CI, 7.9 to 16.5 months)	no data	Ki67 predicted PFS (HR 1.17, 95% CI: 1.03 to 1.33, p-value=0.02)
Faiss 1999 (18)	prospective study	30 patients with metastatic GEP NET, progressive during treatment with somatostatin analogues and/or IFN-a	Ultra-high dose of lanreotide (5mg SC TDS) for one year	33.3% previous SSAs treatment	foregut 20% (6/30), midgut 30% (9/30), hindgut 16.6% (5/10); unknown 33.3% (10/30)	functional midgut NET, 1 complete and 1 partial remission after 12 months; 11 stable; 11 continuing growth after 3-12 months; hindgut tumours: all progressed	90% (27/ 30) of patients	location of tumour
Palazzo et al 2013 (13)	retrospective	68 patients, LAN monotherapy<br>- progressive disease at baseline: 18.5% of cases	LAN (microparticles [MP] 14 days or Autogel 28 days) for minimum 3 months; median dose 90mg every 28 days	SSA naïve	foregut 28% (19/68); midgut 59% (40/68); unknown 13% (9/68)	median PFS 29 months	62/68 (91%) at baseline	Non-response was predicted by Ki-67 >5% (HR 0.262, p=0.009); pre-treatment progressive disease (HR 0.241, p=0.008); hepatic tumour load > 25% (HR 0.237, p=0.004)

Caplin et al 2016 (5)	open label extension study, case control	Patients previously on lanreotide (n=41) and placebo (n=47) (from the CLARINET study)	LAN autogel 120mg every 28 days<br>- continuous LAN: 26-74.3months<br>- PBO-LAN with SD at end of Core study, 1-49.9months<br>- PBO-LAN with PD at end of core study, 2.0-52months	naïve	pancreas 38%; midgut 39%; 23% other/unknown primary tumours	core study to PD in core/OLE: sensitivity analysis, median 30.8 months (95% CI: 30.0 to 31.3); PBO-LAN median time to further PD 14 months (10.1; not reached)	HTL >25-50% LAN-LAN group: 24.4% of cases; PBO-LAN group, no PD at core study (0%), PD during core study (15.6% of cases)	no data
Bianchi 2011 (14)	retrospective	23 patients with well differentiated metastatic NET	LAN 120mg monthly for up to 24 months	21.7% previous SSA treatment	34.8% pancreas, 30.4% bowel	median duration of response 28 months (range 6-50) radiological response: 2 partial regression (8.7%); 15 stable (65.3%), 6 progressed (26%)	78.2% at baseline, no further quantification	no data
Ducreux M 2000 (15)	open, prospective, phase II trial	46 patients with progressive disease (30 symptomatic and 16 non-symptomatic)	symptomatic carcinoid: lanreotide 30mg IM every 14days for 12 months; lanreotide 30mg every 10days if asymptomatic for 13 months	only 7 symptomatic were naïve	intestine 17/30 symptomatic; intestine 6/16 asymptomatic; pancreas 6/16 asymptomatic	radiological stability total of 19/39 patients, 9.5 months; progression total 16/39 patients), event at 3 months (5/39 cases) and at 6 months (11 cases)	no data	none identified
Khan 2011 (16)	retrospective	76 patients with metastatic GEP NET and carcinoid	LAN 60mg/28 days in 23 patients, 90mg/28days in 36 patients, 120mg in 7 patients<br>Median follow up 33 months	11 (16%) had previous octreotide	midgut	radiological PFS± clinical: 93% at year 1, 75% at 3 years, 59% at 5 years; clinical PFS±radiological 94% at year 1, 70% at 3 years and 45% at 5 years.	no data	PFS radiological/clinical differed between those requiring and not requiring addition of second treatment; PFS did not differ between G1 and G2/G3

Supplementary table 1b. Studies evaluating PFS in patients treated with Lanreotide combination regimens								
**Author**	**Type of study**	**Sample size**	**Lanreotide regimen**	**Patients SSA naïve or not**	**Location of tumour**	**PFS**	**Liver disease**	**Predictors of PFS**
Capdevila 2015 (17)	retrospective, cross-sectional	133 patients with metastatic disease at diagnosis (98.5%)	115 patients received LAN combination with everolimus or sunitinib Duration of treatment up to 35.6 months	86.5% prior SSTAs	foregut 64%; midgut 30% 22.5%; hindgut 4.5%)	sunitinib and LAN, PFS 78.5% (6 months); 68.6% (12 months); 73% (12 months); 67.4% (18 months); everolimus plus LAN median PFS 25.8 months (95% CI, 11.3 to 40.3)	no data	no data
Faiss 2003 (12)	prospective, randomized, multicenter study	80 patients with progressive disease, therapy naïve, lanreotide 25 patients, IFN-a 27 patients, lanreotide and IFN-a 28 patients	Lanreotide 1mg TDS vs IFN-a 5million Units three times a week vs both Duration of treatment 12 months	SSA naïve; therapy naïve	45% (36/80) foregut, 37.5% (30/80) midgut, 3.75% (3/80) hindgut, 13.75% (11/80) unkwown; 36.25% (29/80) functional, 63.75% (51/80) nonfunctional	PFS did not differ between regimen types	73 patients (91.3%) at baseline	tumour location
Ida 2018 (19)	retrospective	12 patients with unresectable foregut/hindgut NET	LAN or Octreotide LAR for a median of 25.9 months	naïve to SSAs	rectum 83.3%, stomach 8.3%, duodenum 8.3%	median 13.6months	≤25% 9 patients; >25% 3 patients	PFS was not predicted by G1/G2 staging, hepatic tumour load
Panzuto 2006 (20)	retrospective	31 patients with entero-pancreatic well differentiated NET and progressive metastatic disease	Octreotide LAR 30mg or LAN SR 60mg every 28 days Duration of follow up 6 up to 60 months	SSA naïve all, previous chemotherapy 12.9%, chemoembolization 64.55%	Pancreas 58%; intestine 35.5%; unknown 6.5%	PFS 26.5months 6 months: stabilization pancreatic vs intestinal, 27.8% vs 81.8%; 3 years survival: responders vs non-responders 52.3%	87% liver metastases at baseline	predictors for non-response: pancreatic primary tumour, absence of previous surgery, presence of distant extra-hepatic metastases
Ozaslan 2017 (21)	retrospective	165 patients	Octreotide LAR dose: 96 patients	first line therapy: SSA 104 and CTx	pancreas 31%; GI 30%; lung 16%	SSA PFS 21 months (95% CI: 12.4 to 29.6) and CTx	73% liver metastases at	SSA PFS predictors: i)

			20mg or 30mg and LAN 8 patients 120mg<br>Median follow up: 36 months	61; <u>second line</u> SSA 31 and CTx 34; <u>third line</u>: SSA 11 and CTx 12		PFS 8 months (95% CI: 5.5 to 10.6)	baseline; no further quantification of hepatic involvement	surgical resection; ii) G1 stage; iii) stable disease at initiation of treatment

Abbreviations:

LAN: lanreotide; PFS: progression-free survival; HTL: hepatic tumour load; PBO: placebo; PD: progressive disease; SSA: somatostatin analogue; RT: radiotherapy; CTx: chemotherapy
